# Rescued chlorhexidine activity by resveratrol against carbapenem-resistant *Acinetobacter baumannii* via down-regulation of AdeB efflux pump

**DOI:** 10.1371/journal.pone.0243082

**Published:** 2020-12-02

**Authors:** Uthaibhorn Singkham-in, Paul G. Higgins, Dhammika Leshan Wannigama, Parichart Hongsing, Tanittha Chatsuwan

**Affiliations:** 1 Department of Microbiology, Faculty of Medicine, Chulalongkorn University, Bangkok, Thailand; 2 Antimicrobial Resistance and Stewardship Research Unit, Faculty of Medicine, Chulalongkorn University, Bangkok, Thailand; 3 Institute for Medical Microbiology, Immunology and Hygiene, University of Cologne, Cologne, Germany; 4 German Centre for Infection Research (DZIF), Partner site Bonn-Cologne, Cologne, Germany; 5 School of Medicine, Faculty of Health and Medical Sciences, The University of Western Australia, Nedlands, Western Australia, Australia; 6 Mae Fah Luang University Hospital, Mae Fah Luang University, Chiang Rai, Thailand; 7 School of Integrative Medicine, Mae Fah Luang University, Chiang Rai, Thailand; Emory University School of Medicine, UNITED STATES

## Abstract

The aim of this study was to determine the activity and synergistic mechanisms of resveratrol in combination with chlorhexidine against carbapenem-resistant *Acinetobacter baumannii* clinical isolates. The activity of resveratrol plus antimicrobial agents was determined by checkerboard and time-kill assay against carbapenem-resistant *A*. *baumannii* isolated from patients at the King Chulalongkorn Memorial Hospital, Bangkok, Thailand. Overexpression of efflux pumps that mediates chlorhexidine susceptibility was characterized by the ethidium bromide accumulation assay. The effect of resveratrol on the expression of efflux pump genes (*adeB*, *adeJ*, *adeG abeS*, and *aceI*) and the two-component regulators, *adeR* and *adeS* was determined by RT-qPCR. The combination of resveratrol and chlorhexidine resulted in strong synergistic and bactericidal activity against carbapenem-resistant *A*. *baumannii*. Up-regulation of *adeB* and *aceI* was induced by chlorhexidine. However, the addition of resveratrol increased chlorhexidine susceptibility with increased intracellular accumulation of ethidium bromide in *A*. *baumannii* indicating that resveratrol acts as an efflux pump inhibitor. Expression of *adeB* was significantly reduced in the combination of resveratrol with chlorhexidine indicating that resveratrol inhibits the AdeB efflux pump and restores chlorhexidine effect on *A*. *baumannii*. In conclusion, reduced *adeB* expression in *A*. *baumannii* was mediated by resveratrol suggesting that AdeB efflux pump inhibition contributes to the synergistic mechanism of resveratrol with chlorhexidine. Our finding highlights the potential importance of resveratrol in clinical applications.

## Introduction

*Acinetobacter baumannii* has become an important opportunistic pathogen in healthcare settings worldwide [1]. Various nosocomial infections such as pneumonia (particularly ventilator-associated), wound, and bloodstream infection caused by *A*. *baumannii* are associated with significantly increased mortality in hospitalized patients particularly those in ICU settings [2, 3]. However, community-acquired infections caused by *A*. *baumannii* are also linked to critical mortality rates due to patient’s risk factors including alcoholism, smoking, and lung disease [4]. The challenge for the treatment of *A*. *baumannii* infection is antimicrobial resistance. The emergence of multidrug-resistant *A*. *baumannii* (resistant to commonly used antimicrobial agents including aminoglycosides, fluoroquinolones, extended-spectrum cephalosporins, and carbapenems) has increasingly been reported around the world [5]. Despite polymyxins and tigecycline being used as the last-resort drugs for the treatment of carbapenem-resistant *A*. *baumannii* infection, widespread resistance to these antibiotics was globally found [6, 7]. This highlights the limitations for the treatment of *A*. *baumannii* infection especially carbapenem-resistant strains. Combination therapies have therefore become one of the options for treatment, however, *in vitro* activity of antibiotic combinations do not strongly correlate with clinically relevant outcome in patients [8, 9].

Recently, carbapenem-resistant *A*. *baumannii* was classified by the World Health Organization (WHO) as a critical pathogen that needs novel antibiotics for treatment [10]. Unfortunately, the discovery rate of new antibiotics is not correlated with antibiotic resistance rates of superbugs, especially *A*. *baumannii*. Novel therapies using bacteriophages, antimicrobial peptides, and novel natural compounds for the treatment of *A*. *baumannii* infections are interesting. For example natural compounds extracted from plants especially flavonoids, including curcumin and epigallocatechin gallate (EGCG) have potential activity on biofilm formation and reduced virulence of multidrug-resistant *A*. *baumannii* [11].

Similarly, resveratrol (3,5,4′-trihydroxy-trans-stilbene) is a natural compound extracted from grape skin and seeds [12]. It is a phytoalexin belonging to the polyphenol stilbenoids group, that displays considerable antimicrobial activity against diverse human pathogens, including bacteria [13, 14]. Resveratrol can alter bacteria virulence, reduce membrane integrity, and inhibit biofilm formation via interference of quorum sensing [13–15]. Moreover, resveratrol is safe for humans and has been used as a food preservative due to its antimicrobial and antibiofilm properties against foodborne pathogens [14, 16]. Here we aim to investigate the enhancement effect of resveratrol by determining the activity of resveratrol alone, and in combination with other antimicrobial agents against carbapenem-resistant *A*. *baumannii*.

## Materials and methods

### Bacterial isolates and susceptibility testing

A total of 20 carbapenem-resistant *A*. *baumannii* clinical isolates with various morphology and resistance profiles were obtained without preference from a strain repository at the Department of Microbiology, King Chulalongkorn Memorial Hospital. Clinical isolates used in this study had been isolated during 2016–2018 from infected patients as part of standard care of the patients and were unrelated to the present study. Susceptibility to imipenem (Apollo Scientific, UK), colistin, rifampicin, chlorhexidine, and resveratrol (Sigma-Aldrich, Germany) was determined by broth microdilution method according to the Clinical and Laboratory Standards Institute (CLSI) guidelines [17]. Resveratrol solution in all experiments was prepared in 0.5% v/v DMSO. *Escherichia coli* ATCC 25922 and *Pseudomonas aeruginosa* ATCC 27853 were used as control strains for susceptibility testing. This study was approved by the Institutional Review Board of the Faculty of Medicine, Chulalongkorn University, Bangkok, Thailand (IRB 571/61).

### Detection of carbapenemase genes

Presence of OXA-type carbapenemase genes including *bla*_OXA-51-like_, *bla*_OXA-23-like_, *bla*_OXA-24/40-like_, *bla*_OXA-58-like_, *bla*_OXA-143-like_, and *bla*_OXA-235-like_ was determined by multiplex PCR as described by Higgins et al. [18]. Presence of metallo-carbapenemase genes including *bla*_IMP-like_, *bla*_VIM-like_, *bla*_GIM-like_, and *bla*_SPM-like_ was investigated by multiplex PCR as described by Ellington et al. [19]. A further multiplex PCR described by Poirel et al. [20] was used to detect *bla*_OXA-48-like_, *bla*_NDM-like_, and *bla*_KPC-like_.

### Checkerboard assay

*In vitro* activity of resveratrol in combination with imipenem, colistin, rifampicin, and chlorhexidine was performed in 96-well microtiter plate by checkerboard assay as previously described [21]. Briefly, each well in the row of the plates contained cation-adjusted Mueller-Hinton agar (Difco, USA) supplemented with two-fold serial dilution of resveratrol. Two-fold serial dilution of imipenem, colistin, rifampicin, or chlorhexidine was added in the column of the plates. After the inoculation of *A*. *baumannii*, the plates were incubated at 35°C for 20–24 h. Fractional inhibitory concentration index (FICI) was calculated and interpreted as follows: ≤0.5 = synergy, >4 = antagonism and >0.5–4 = no interaction [22].

### Time-kill assay

The combination of resveratrol and imipenem, colistin, rifampicin, or chlorhexidine which showed the highest synergy was further investigated by time-kill assay as previously described [21]. Briefly, viable cells of *A*. *baumannii* in each following condition: growth control, DMSO (Sigma-Aldrich, Germany) control, 128 mg/L of resveratrol, 0.25× MIC of chlorhexidine, and the combination of resveratrol and chlorhexidine was collected and quantified after 0, 2, 4, 6, 8, and 24 h of incubation at 35°C with shaking at 120 rpm. Synergism and bactericidal activity were defined as the reduction of viable cell ≥2log_10_ (CFU/mL)-fold compared with single-agent, and ≥3log_10_ (CFU/mL)-fold reduction compared with the viable cell at start time point, respectively. The time-kill assay was done independently in triplicate.

### Ethidium bromide accumulation assay

The accumulation of ethidium bromide was performed as described by Nowak et al. [23] with a slight modification. Briefly, mid-log phase bacterial cultures were harvested by centrifugation, washed twice, and resuspended in phosphate buffer saline to an optical density (OD) of 0.2 at 600 nm. The bacterial suspension was transferred to a black 96-well microtiter plate. Glucose and ethidium bromide were added to the suspension at final concentration of 0.2% (w/v) and 10 μM, respectively. The fluorescence of ethidium bromide was measured (excitation 535 nm and emission 590 nm) every 40 seconds for 1600 seconds by using Varioskan Flash Multimode Reader (Thermo Fisher Scientific, USA). Another experiment was performed as described above by adding CCCP or resveratrol (at final concentration of 500 μM and 128 mg/L, respectively) after measuring fluorescence for 800 seconds. The fluorescence was measured for another 800 seconds. Moreover, the accumulation assay was performed in the presence of chlorhexidine at final concentration of 8 mg/L. These experiments were done independently in triplicate.

### Expression of efflux pump genes

The presence of transcripts encoding the efflux pump genes, *adeB*, *adeJ*, *adeG*, *abeS*, *aceI*, and the *adeABC* two-component regulators *adeR* and *adeS* were detected by qPCR assay using primers listed in S1 Table. Mid-log phase *A*. *baumannii* isolate L14 was treated with DMSO, 128 mg/L of resveratrol, 0.25× MIC of chlorhexidine, or the combination of resveratrol and chlorhexidine for 10 min. After treatment, total RNA of collected cells was extracted by using Monarch total RNA miniprep kit (NEB, USA). SuperScript^®^ III reverse transcriptase (Thermo Fisher Scientific, USA) was used to convert total mRNA to cDNA. RT-qPCR was performed by using Luna^®^ Universal qPCR master mix (NEB, USA) and QuantStudio5 (Thermo Fisher Scientific, USA). Relative expression level of efflux pump genes was calculated by using 16S rRNA as a reference gene. The RT-qPCR experiments were performed in at least three independent experiments. Differences of the relative expression level in efflux pump genes in each treatment were determined by using analysis of variance (ANOVA) (#, *p*-value<0.1; *, *p*-value <0.05; **, *p*-value <0.01; ***, *p*-value <0.001 were considered to be significant).

## Results

### Antimicrobial susceptibility and carbapenemase encoding genes in *A*. *baumannii*

The results of antimicrobial susceptibility testing are shown in Table 1. All 20 *A*. *baumannii* isolates were resistant to imipenem, with the MIC range of 32 to 128 mg/L. All isolates were susceptible to colistin (MIC range, 0.5 to 2 mg/L). Although rifampicin is not commonly used for treatment and no interpretation criteria for *A*. *baumannii* is provided, 75% of isolates showed low MICs to rifampicin from 1 to 4 mg/L. Fifteen isolates (75%) were highly resistant to amikacin (MIC >256 mg/L), whereas five isolates (25%) were susceptible to amikacin with MIC range of 0.5 to 16 mg/L. The MIC range of the antiseptic agent, chlorhexidine, was 16 to 32 mg/L. There is no standard interpretation for chlorhexidine susceptibility in *A*. *baumannii*. However, the MIC range of *A*. *baumannii* clinical isolated has been reported worldwide to be is 8–400 mg/L [24]. Resveratrol showed no antimicrobial activity against *A*. *baumannii* (MIC >512 mg/L). All of the *A*. *baumannii* isolates co-carried the intrinsic carbapenemase gene, *bla*_OXA-51-like_ with an acquired carbapenemase gene, *bla*_OXA-23-like_ (Table 1).

**Table 1:** MICs of antibiotics, chlorhexidine, and resveratrol of 20 *A*. *baumannii* isolates.

Strain	Carbapenemase gene	MIC (mg/L)					
		Imipenem	Colistin	Rifampicin	Chlorhexidine	Amikacin	Resveratrol
AC151	*bla*_OXA-51_ + *bla*_OXA-23_	64	1	4	32	2	>512
AC152	*bla*_OXA-51_ + *bla*_OXA-23_	128	2	2	16	>256	>512
AC153	*bla*_OXA-51_ + *bla*_OXA-23_	32	1	>256	32	>256	>512
AC154	*bla*_OXA-51_ + *bla*_OXA-23_	128	1	>256	32	>256	>512
AC155	*bla*_OXA-51_ + *bla*_OXA-23_	128	1	4	32	>256	>512
AC156	*bla*_OXA-51_ + *bla*_OXA-23_	128	1	4	32	>256	>512
AC157	*bla*_OXA-51_ + *bla*_OXA-23_	128	1	4	32	>256	>512
AC158	*bla*_OXA-51_ + *bla*_OXA-23_	64	1	4	16	2	>512
AC159	*bla*_OXA-51_ + *bla*_OXA-23_	128	1	4	32	>256	>512
AC160	*bla*_OXA-51_ + *bla*_OXA-23_	64	2	4	32	>256	>512
L25	*bla*_OXA-51_ + *bla*_OXA-23_	32	1	1	32	0.5	>512
L26	*bla*_OXA-51_ + *bla*_OXA-23_	64	1	4	32	>256	>512
L27	*bla*_OXA-51_ + *bla*_OXA-23_	64	2	256	32	>256	>512
L28	*bla*_OXA-51_ + *bla*_OXA-23_	64	2	64	32	>256	>512
L29	*bla*_OXA-51_ + *bla*_OXA-23_	64	1	64	32	>256	>512
L12	*bla*_OXA-51_ + *bla*_OXA-23_	32	1	4	32	16	>512
L14	*bla*_OXA-51_ + *bla*_OXA-23_	32	0.5	2	32	0.5	>512
L20	*bla*_OXA-51_ + *bla*_OXA-23_	128	1	4	32	>256	>512
L21	*bla*_OXA-51_ + *bla*_OXA-23_	128	1	4	32	>256	>512
L23	*bla*_OXA-51_ + *bla*_OXA-23_	128	1	4	32	>256	>512

### Activity of resveratrol in combination with antimicrobial agents

Since resveratrol exhibited no antimicrobial activity, we determined the *in vitro* activity of resveratrol in combination with imipenem, colistin, rifampicin, and chlorhexidine by checkerboard assay. The FICIs of resveratrol in combination with antimicrobial agents are shown in Table 2. No synergism of resveratrol plus imipenem, colistin, or amikacin was observed. Resveratrol with rifampicin had a synergistic effect on three (15%) carbapenem-resistant *A*. *baumannii* isolates. Interestingly, the most effective combination was resveratrol plus chlorhexidine. This combination had synergism against all 20 carbapenem-resistant *A*. *baumannii* isolates. None of the combinations showed antagonism.

**Table 2:** FICIs of resveratrol in combination with antibiotics or chlorhexidine in 20 *A*. *baumannii* isolates.

Strain	FICI (interpretation)				
	Resveratrol + Imipenem	Resveratrol + Colistin	Resveratrol + Rifampicin	Resveratrol + Chlorhexidine	Resveratrol + Amikacin
AC151	2 (N)	0.53 (N)	0.56 (N)	0.38 (S)	0.75 (N)
AC152	2 (N)	2 (N)	0.31 (S)	0.19 (S)	1.5 (N)
AC153	2 (N)	2 (N)	2 (N)	0.31 (S)	1.5 (N)
AC154	2 (N)	2 (N)	0.56 (N)	0.19 (S)	0.75 (N)
AC155	0.52 (N)	2 (N)	0.31 (S)	0.31 (S)	1.5 (N)
AC156	2 (N)	2 (N)	0.52 (N)	0.31 (S)	1.5 (N)
AC157	2 (N)	2 (N)	0.52 (N)	0.31 (S)	1.5 (N)
AC158	2 (N)	2 (N)	0.51 (N)	0.31 (S)	2 (N)
AC159	2 (N)	2 (N)	0.51 (N)	0.19 (S)	2 (N)
AC160	2 (N)	0.52 (N)	0.31 (S)	0.38 (S)	2 (N)
L25	0.56 (N)	1 (N)	0.52 (N)	0.31 (S)	2 (N)
L26	2 (N)	2 (N)	2 (N)	0.31 (S)	2 (N)
L27	0.56 (N)	0.63 (N)	2 (N)	0.31 (S)	2 (N)
L28	2 (N)	0.63 (N)	0.53 (N)	0.31 (S)	2 (N)
L29	2 (N)	2 (N)	0.56 (N)	0.19 (S)	1 (N)
L12	0.56 (N)	0.56 (N)	2 (N)	0.31 (S)	2 (N)
L14	0.56 (N)	2 (N)	0.51 (N)	0.38 (S)	2 (N)
L20	2 (N)	0.63 (N)	0.52 (N)	0.19 (S)	2 (N)
L21	2 (N)	2 (N)	0.51 (N)	0.19 (S)	1 (N)
L23	2 (N)	2 (N)	0.51 (N)	0.19 (S)	0.75 (N)

### Killing activity of resveratrol in combination with chlorhexidine against *A*. *baumannii*

**Fig 1:**
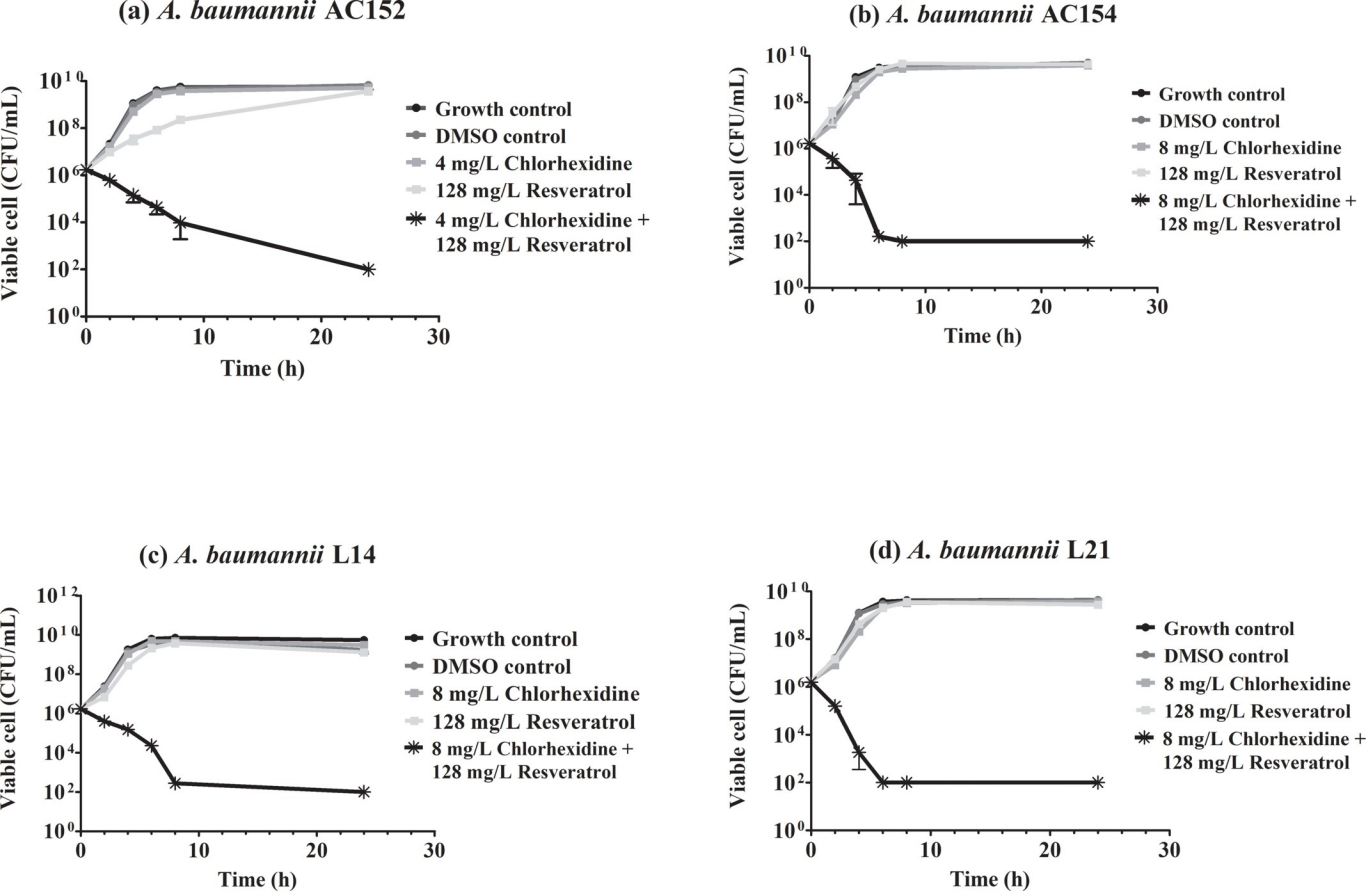
Time-kill curves of resveratrol and chlorhexidine against *A*. *baumannii*. Time-kill curves of resveratrol 128 mg/L in combination with 0.25x MIC of chlorhexidine (4 or 8 mg/L) against *A*. *baumannii* strain AC152 (a), AC154 (b), L14 (c), and L21(d). Mean values of viable cells were plotted with error bars representing standard deviations. All experiments were performed in triplicate and the detection limit of the viable cells is 10^2^ CFU/mL.

Due to the finding that resveratrol with chlorhexidine was the most effective combination in our study, we further investigated its synergism by time-kill study. According to the synergism by checkerboard assay, the combination of 128 mg/L of resveratrol and 0.25× MIC of chlorhexidine was performed against four carbapenem-resistant *A*. *baumannii* isolates which differed in antimicrobial susceptibility. Time-kill curves of four *A*. *baumannii* isolates (AC152, AC154, L14, and L21) are shown in Fig 1. Resveratrol alone (128 mg/L) did not inhibit the growth of *A*. *baumannii* isolates. This is similar to chlorhexidine alone at a sub-inhibitory concentration (0.25× of MIC), which could not inhibit the growth of *A*. *baumannii*. Interestingly, the combination of resveratrol and chlorhexidine at these concentrations dramatically decreased *A*. *baumannii* viable cells greater than 2log_10_ (CFU/mL)-fold compared to those of single-agent indicating synergism. After 24 hr of incubation, the combination of resveratrol and chlorhexidine resulted in reduced viable cells with greater than 3log_10_ (CFU/mL)-fold compared to those at a start time point indicating the bactericidal effect of this combination.

### Increased ethidium bromide accumulation in *A*. *baumannii* by CCCP and resveratrol

**Fig 2:**
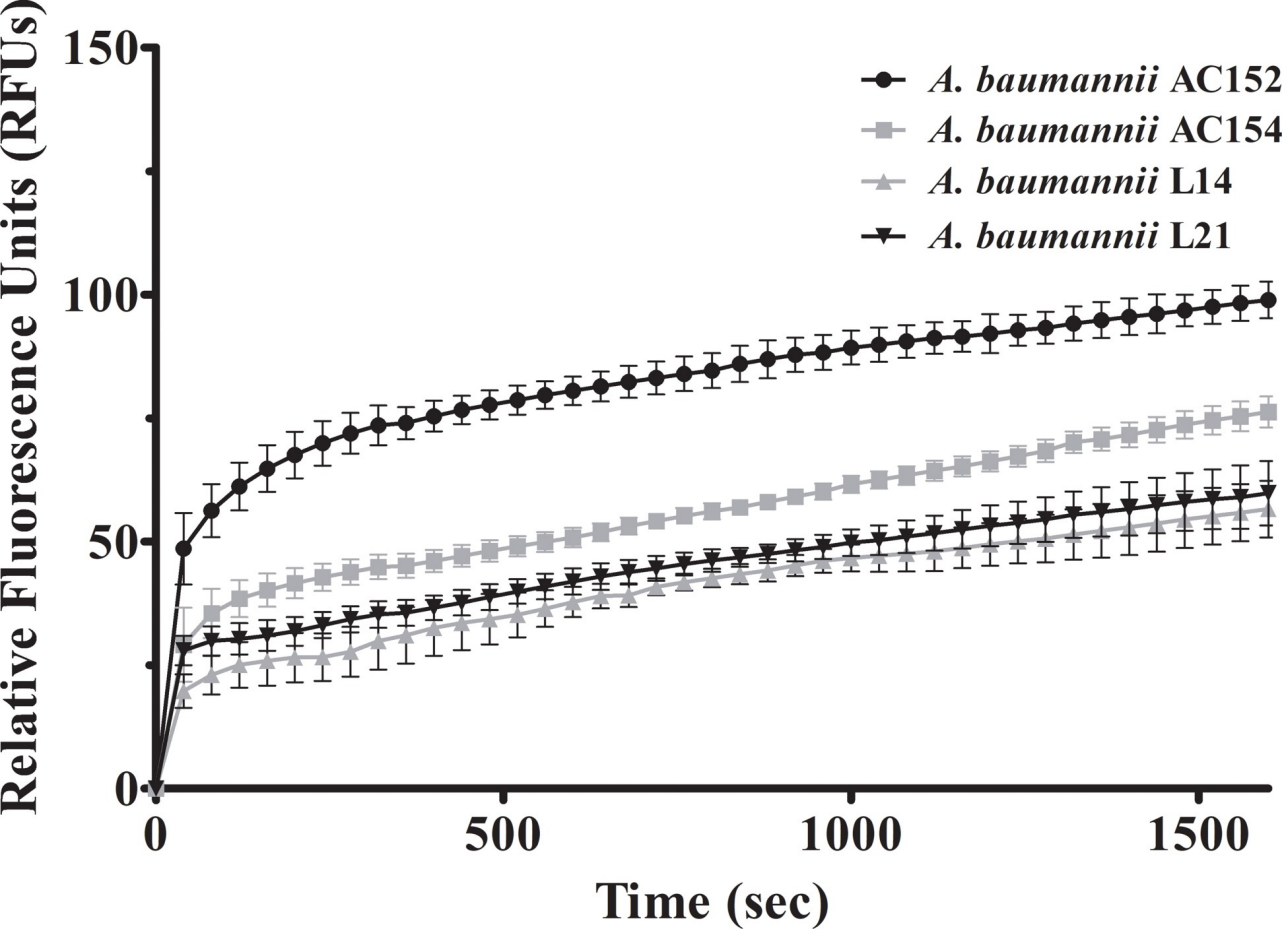
Accumulation of ethidium bromide in *A*. *baumannii* strain AC152, AC154, L14, and L21. The fluorescence representing ethidium bromide accumulation was measured in the presence of glucose as an energy source every 40 seconds for 1600 seconds. Relative fluorescence units were plotted with error bars representing standard deviations. All experiments were performed in triplicate.

**Fig 3:**
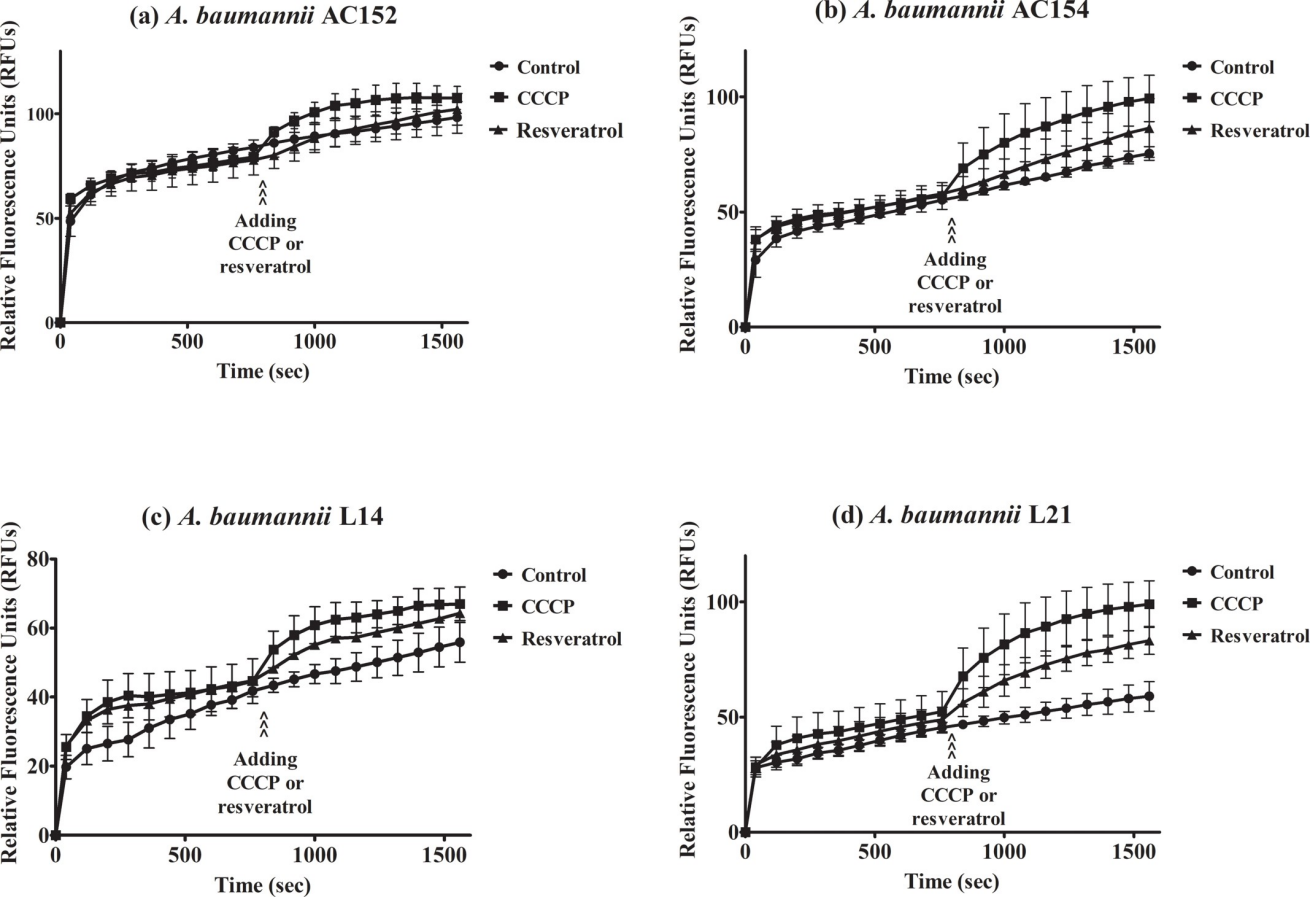
Effect of resveratrol on ethidium bromide accumulation in *A*. *baumannii* strain AC152 (a), AC154 (b), L14 (c), and L21 (d). The fluorescence of ethidium bromide was measured in the presence of glucose and after addition of CCCP (filled squares), resveratrol (filled triangles), or control with no addition of any proton coupler agent (fill circles). Relative fluorescence units were plotted with error bars representing standard deviations. All experiments were performed in triplicate.

**Fig 4:**
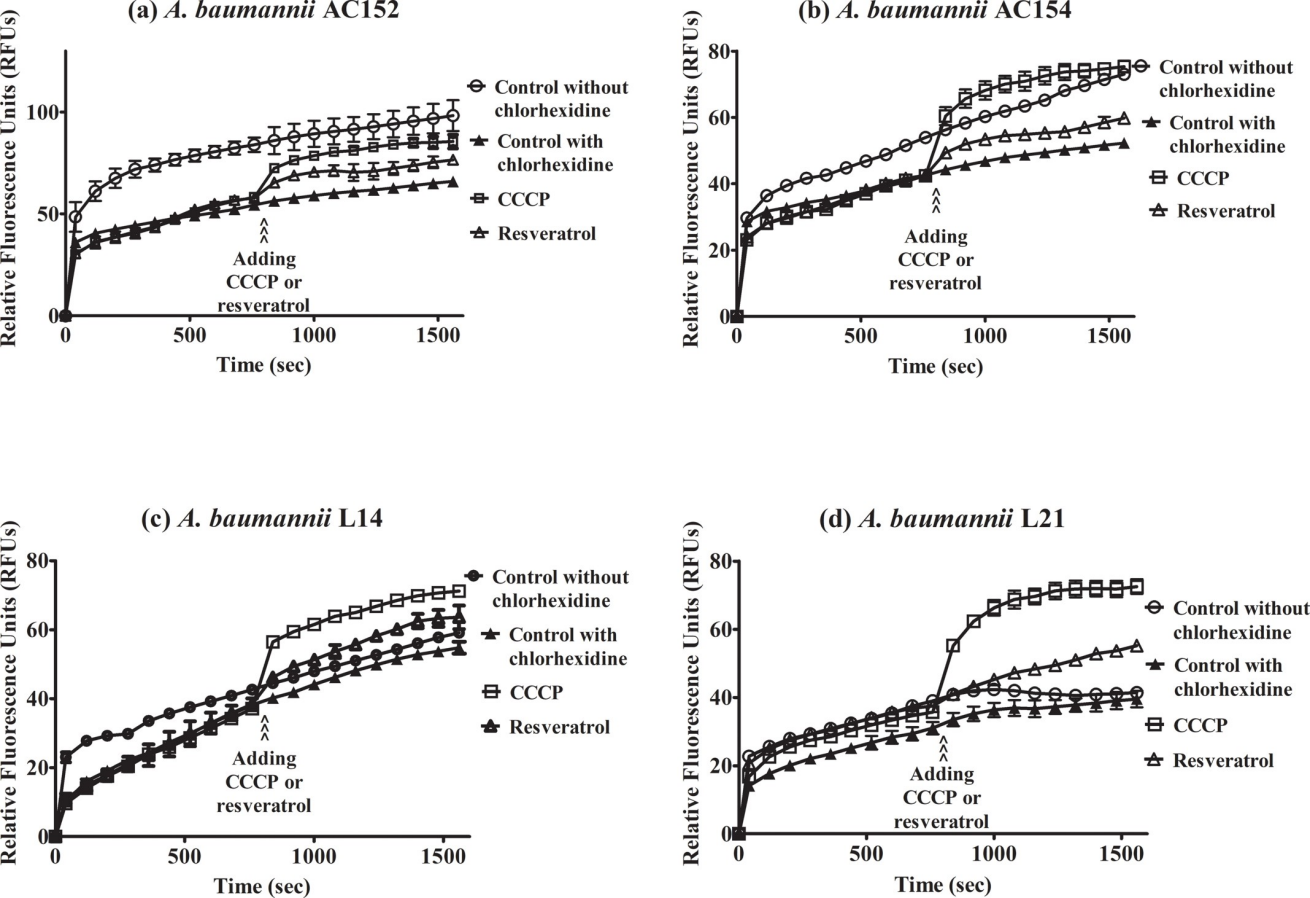
Effect of resveratrol in the presence of chlorhexidine on ethidium bromide accumulation in *A*. *baumannii* strain AC152 (a), AC154 (b), L14 (c), and L21 (d). The fluorescence of ethidium bromide was measured in the presence of glucose with (filled triangles) or without (open circles) chlorhexidine (8 mg/L) and after addition of CCCP (open squares) or resveratrol (open triangles). Relative fluorescence units were plotted with error bars representing standard deviations. All experiments were performed in triplicate.

CCCP is a proton conductor which interrupts the efflux pump function by de-energizing cells. To verify whether resveratrol affects the function of efflux pumps, we performed an assay to detect the accumulation of ethidium bromide. The levels of the relative fluorescence unit (RFU) are proportional to the ethidium bromide accumulation. The levels of fluorescence were increased in carbapenem-resistant *A*. *baumannii* during 1600 seconds indicating accumulation of ethidium bromide over time till saturation (Fig 2), with the highest accumulation as approximately 98 RFUs observed in *A*. *baumannii* AC152, followed by *A*. *baumannii* AC154, L21, and L14 at approximately 76, 59, and 56 RFUs, respectively. Addition of CCCP after 800 seconds of detection resulted in the raising of accumulated ethidium bromide (Fig 3). Increased ethidium bromide accumulation was also observed after resveratrol addition (Fig 3) indicating that resveratrol affects efflux pumps. Moreover, the accumulation assay was performed in the presence of chlorhexidine to investigate the effect of chlorhexidine on ethidium bromide accumulation. Ethidium bromide accumulations in the presence of chlorhexidine were lower than those in the absence of chlorhexidine (Figs 3 and 4) indicating that chlorhexidine increased efflux pump activity. Furthermore, the addition of CCCP or resveratrol in the presence of chlorhexidine led to increased ethidium bromide accumulation (Fig 4) indicating that CCCP or resveratrol may inhibit chlorhexidine efflux pumps.

### Effect of resveratrol on efflux pump expression

**Fig 5:**
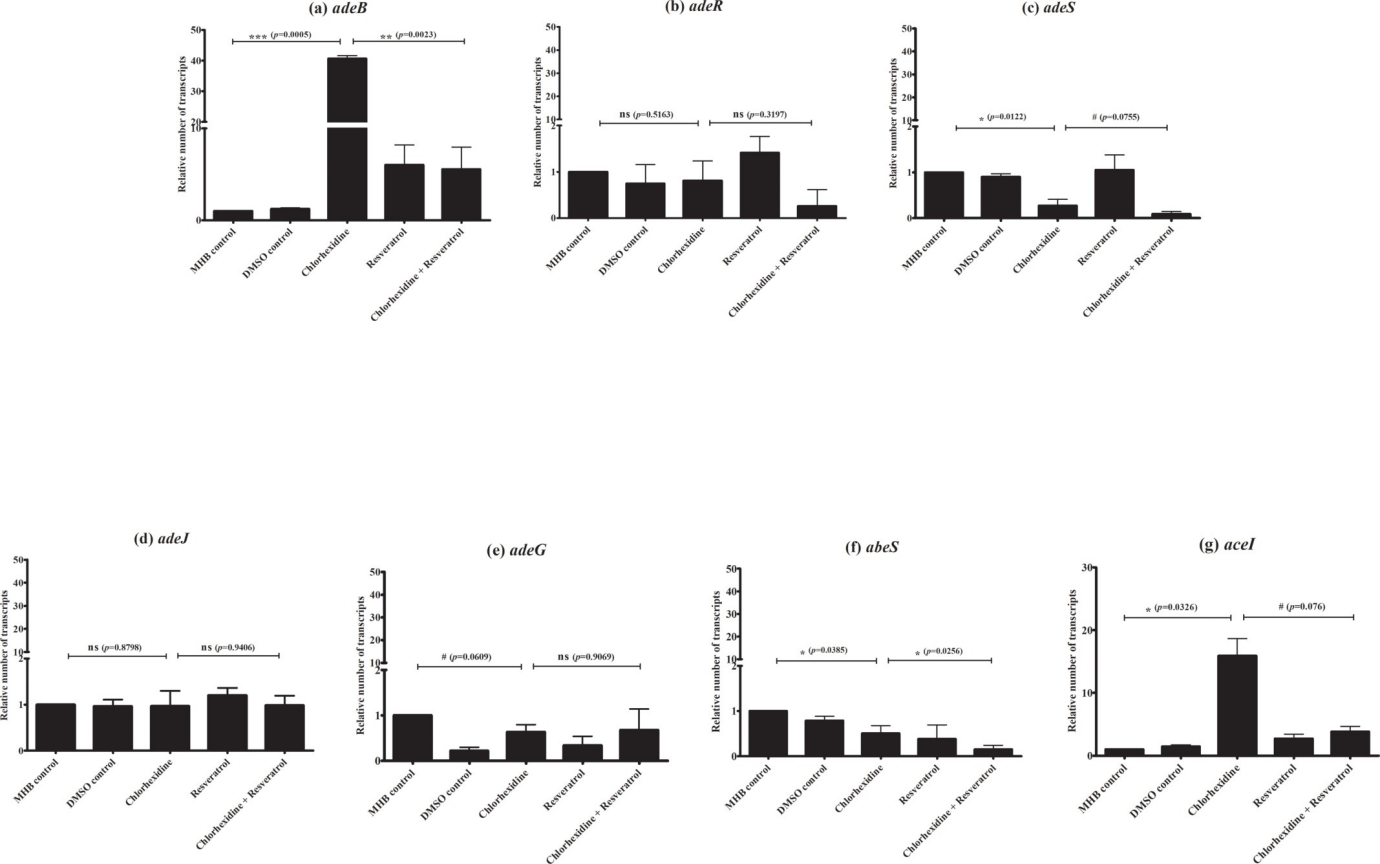
Effect of chlorhexidine and resveratrol alone or in combination on expression of efflux pump and efflux pump regulator genes in *A*. *baumannii* strain L14. RT-qPCR assay of *adeB* (a), *adeR* (b), *adeS* (c), *adeJ* (d), *adeG* (e), *abeS* (f), and *aceI* (g) expression in the presence of MHB control, DMSO control, either chlorhexidine (8 mg/L) or resveratrol (128 mg/L) and in the combination of chlorhexidine with resveratrol. Relative number of transcripts of each gene was normalized to 16S rRNA expression in each condition and calculated using the 2^-ΔΔct^ method compared to the expression level in MHB control. The relative number of transcripts of each gene was plotted with error bars representing standard deviations. All experiments were performed in triplicate. *p-*values were calculated using ANOVA (#, *p*-value <0.1; *, *p-*value <0.05; **, *p-*value <0.01; ***, *p-*value <0.001 and ns = non significant).

Our results suggest that resveratrol affects efflux pumps which are involved in chlorhexidine susceptibility. To determine which efflux pump is associated with chlorhexidine, we determined the effect of 0.25× MIC chlorhexidine (8 mg/L) on efflux gene expression. Chlorhexidine significantly up-regulated *adeB* (*p-*value <0.001) and *aceI* (*p*-value <0.05) expression (Fig 5A and 5G) but no up-regulation of *adeJ*, *adeG* and *abeS* was observed (Fig 5D–5F). Our result confirms that AdeB and AceI efflux pumps are associated with chlorhexidine. To evaluate which efflux pump gene is inhibited by resveratrol, we detected efflux pump gene expression in the presence of both chlorhexidine (8 mg/L) and resveratrol (128 mg/L). Resveratrol more significantly down-regulated *adeB* expression (*p*-value <0.001) in the presence of chlorhexidine (Fig 5A) than *aceI* expression (Fig 5G). This result indicates that resveratrol inhibits AdeB rather than AceI efflux pump. Due to the expression of AdeABC pump associated with the two-component regulate genes, *adeR* and *adeS*, we investigated the expression of *adeR* and *adeS*. However, no overexpression of *adeR* and *adeS* was observed (Fig 5B and 5C) in the presence of chlorhexidine which up-regulated *adeB* expression. Moreover, resveratrol did not significantly down-regulate *adeR* and *adeS* in the presence of chlorhexidine (Fig 5B and 5C). These data indicate that resveratrol enhances chlorhexidine susceptibility by down-regulation of AdeB efflux pump but not via the AdeRS regulatory system.

## Discussion

Outbreaks of carbapenem-resistant *A*. *baumannii*, a serious crisis worldwide including Thailand, are predominantly mediated carbapenemase production (particularly OXA-23) and slightly associated with overexpression of efflux pumps and loss of porins [5, 21, 25]. Due to the limitation of treatment of carbapenem-resistant *A*. *baumannii* infection, colistin is becoming frequently used as a last alternative. No colistin-resistant *A*. *baumannii* was found in our study and colistin-resistant strains have rarely been recovered among carbapenem-resistant *A*. *baumannii* isolated from Thailand [26]. Nevertheless, the emergence of this critical pathogen, carbapenem-resistant *A*. *baumannii* requires new active agents for treatment. In this study, we investigated the activity of resveratrol, a natural compound found in red grapes.

Resveratrol has recently gotten attention due to its anti-oxidant, anti-aging, and anti-cancer activities [12]. Besides those activities, antimicrobial activity of resveratrol has been mentioned against food pathogens, *Campylobacter* spp. and *Listeria monocytogenes* with resveratrol MICs of 50–200 mg/L [13, 14]. In contrast, no antimicrobial activity of resveratrol was observed in our study against *A*. *baumannii* (MIC>512 mg/L). Furthermore, a high level of resveratrol MICs (250 to >1000 mg/L) was reported against other nosocomial pathogens including *Klebsiella pneumoniae* and *P*. *aeruginosa* [27]. Thus, resveratrol alone has narrow antimicrobial activity against these Gram-negative nosocomial pathogens.

Interestingly, resveratrol could restore chlorhexidine MICs (reduced >4-fold of MIC) and provided synergism with chlorhexidine against *A*. *baumannii*. Reduced chlorhexidine susceptibility in *A*. *baumannii* mediated by extruding chlorhexidine outside the cells via various multidrug efflux pumps [28]. We hypothesized that efflux pump expression is affected by exposure to resveratrol leading to increased chlorhexidine susceptibility. Evidence for this was demonstrated by CCCP or resveratrol reducing ethidium bromide accumulation. Moreover, lower levels of ethidium bromide accumulation in the presence of chlorhexidine were observed. Taken together, these results strongly suggest that efflux pumps associated with reduced chlorhexidine susceptibility are inhibited by resveratrol.

AdeABC is a well-characterized efflux pump which mediates resistance to aminoglycosides, tigecycline, and antiseptics such as chlorhexidine [29–31]. Previous transcriptomic study to identify chlorhexidine resistance determinant in *A*. *baumannii* ATCC 17978 revealed that overexpressions of *adeAB* and *aceI* were induced by chlorhexidine [32]. Similar to our result, the sub-inhibitory concentration of chlorhexidine (0.25× MIC) significantly induced the expression of both *adeB* and *aceI*. Although *adeB* expression was strongly induced by chlorhexidine, no up-regulation of *adeRS* was observed. In another study, transcriptomic analysis of inactivation *adeR* (*ΔadeR*) *A*. *baumannii* ATCC 17978 showed that *adeB* transcript was detected in this strain indicating that other regulatory systems are involved in the expression of AdeB pump [33]. This could be explained that up-regulated *adeB* expression by chlorhexidine may be independent of AdeRS. Due to amikacin known as AdeB substrate, we determined the activity of resveratrol in combination with amikacin but no synergism was observed (Table 2). However, most of our *A*. *baumannii* isolates were highly resistant to amikacin (>256 mg/L) and carried aminoglycoside-modifying enzyme (AME) gene, *armA* (S2 Table). It is possible that in our isolates, AME production is the major mechanism of resistance and has a greater effect than AdeB expression on aminoglycoside resistance. AdeIJK efflux pump mediates resistance to biocides including chlorhexidine but less effective than AdeABC [30, 34, 35]. Although AdeFGH is an efflux pump which mediated chlorhexidine susceptibility, no difference of *adeG* expression was observed in chlorhexidine-susceptible and chlorhexidine–resistance *A*. *baumannii* clinical isolates [36]. AbeS, single inner membrane protein, is the smallest efflux pump in *A*. *baumannii* which mediated resistance to especially erythromycin and chloramphenicol, but the deletion of *abeS* had a weak effect on chlorhexidine susceptibility [37]. Taken all together with our results, these suggest that efflux pumps associated with chlorhexidine susceptibility are strain-specific and chlorhexidine up-regulated *adeB* and *aceI* expression. In the combination, rescued chlorhexidine activity by resveratrol correlated with *adeB* expression which was strongly reduced in the presence of chlorhexidine and resveratrol indicating that resveratrol rescued chlorhexidine activity by reduction of *adeB* expression. The role of *adeRS* in *adeB* expression and the responsibility of *adeB* for the efflux of chlorhexidine and its synergy with resveratrol should be determined in *adeRS* knockout and *adeB* knockout strains, respectively. Unfortunately, the construction of these knockout in *A*. *baumannii* clinical isolates is quite difficult due to the recalcitrant nature of the clinical isolates. This is the limitation of our study.

In conclusion, resveratrol alone has no antimicrobial activity but provides synergistic and bactericidal effects in the combination with chlorhexidine against carbapenem-resistant *A*. *baumannii* clinical isolates. Resveratrol restores chlorhexidine activity by down-regulation of AdeB pump which is associated with chlorhexidine susceptibility in *A*. *baumannii*.

## Supporting information

S1 Table Oligonucleotide sequences of primers used for RT-qPCR. (PDF)

S2 Table Characteristics of *A*. *baumannii* clinical isolates. (PDF)
