# Correction: Rescued chlorhexidine activity by resveratrol against carbapenem-resistant *Acinetobacter baumannii* via down-regulation of AdeB efflux pump

**DOI:** 10.1371/journal.pone.0272881

**Published:** 2022-08-04

**Authors:** Uthaibhorn Singkham-in, Paul G. Higgins, Dhammika Leshan Wannigama, Parichart Hongsing, Tanittha Chatsuwan

In the Funding statement, the grant number from the funder Ratchadapisek Somphot Fund for Postdoctoral Fellowship is missing. The grant number is: RA62/013.
