# Supplementary material for: Rescued chlorhexidine activity by resveratrol against carbapenem-resistant Acinetobacter baumannii via down-regulation of AdeB efflux pump
Source: PLoS One. 2020 Dec 2;15(12):e0243082. doi: 10.1371/journal.pone.0243082 (PMC7710055; doi:10.1371/journal.pone.0243082)
Supplement: S1 Table — (PDF) [file pone.0243082.s001.pdf]

S1 Table. Oligonucleotide sequences of primers used for RT-qPCR.

Gene	Primer sequences	Reference
adeB	F- TTAACGATAGCGTTGTAACC	[1]
	R- TGAGCAGACAATGGAATAGT	
adeR	F- ACTACGATATTGGCGACATT	[1]
	R- GCGTCAGATTAAGCAAGATT	
adeS	F- TTGGTTAGCCACTGTTATCT	[1]
	R- AGTGGACGTTAGGTCAAGTT	
adeJ	F- CAACGCTTGCAGTTCCAGTA	This study
	R- CCAGAAATCTGCTGCATTGA	
adeG	F- TTCATCTAGCCAAGCAGAAG	[2]
	R- GTGTAGTGCCACTGGTTACT	
abeS	F- ACGGTTGTGGGTTATGCAGT	This study
	R- AACCAATACAGGCAGCCAAG	
aceI	F- TGACCGGAACACTTGGTGTA	this study
	R- GAACAGTCGCAATCAGCAAA	
16S rRNA	F- GGAGGAAGGTGGGGATGACG	[1]
	R- ATGGTGTGACGGGCGGTGTG	

References

1. Hou PF, Chen XY, Yan GF, Wang YP, Ying CM. Study of the correlation of imipenem resistance with efflux pumps AdeABC, AdeIJK, AdeDE and AbeM in clinical isolates of *Acinetobacter baumannii*. Chemotherapy. 2012;58(2):152-8. http://doi: 10.1159/000335599. Epub 2012 May 16. PMID: 22614896

2. He X, Lu F, Yuan F, Jiang D, Zhao P, Zhu J, Cheng H, Cao J, Lu G. Biofilm formation caused by clinical *Acinetobacter baumannii* isolates is associated with overexpression of the AdeFGH efflux pump. Antimicrob Agents Chemother. 2015 Aug;59(8):4817-25. http://doi: 10.1128/AAC.00877-15. PMID: 26033730
