# Supplementary material for: Rescued chlorhexidine activity by resveratrol against carbapenem-resistant Acinetobacter baumannii via down-regulation of AdeB efflux pump
Source: PLoS One. 2020 Dec 2;15(12):e0243082. doi: 10.1371/journal.pone.0243082 (PMC7710055; doi:10.1371/journal.pone.0243082)
Supplement: S2 Table — (PDF) [file pone.0243082.s002.pdf]

S2 Table. Characteristics of *A. baumannii* clinical isolates.

Strain	Carbapenemase gene	Aminoglycoside-modifying enzyme gene	MIC (mg/L)			
			Imipenem	Chlorhexidine	Amikacin	Resveratrol
AC151	$bla_{OXA-51} + bla_{OXA-23}$	-	64	32	2	>512
AC152	$bla_{OXA-51} + bla_{OXA-23}$	*armA*	128	16	>256	>512
AC153	$bla_{OXA-51} + bla_{OXA-23}$	*armA*	32	32	>256	>512
AC154	$bla_{OXA-51} + bla_{OXA-23}$	*armA*	128	32	>256	>512
AC155	$bla_{OXA-51} + bla_{OXA-23}$	*armA*	128	32	>256	>512
AC156	$bla_{OXA-51} + bla_{OXA-23}$	*armA*	128	32	>256	>512
AC157	$bla_{OXA-51} + bla_{OXA-23}$	*armA*	128	32	>256	>512
AC158	$bla_{OXA-51} + bla_{OXA-23}$	-	64	16	2	>512
AC159	$bla_{OXA-51} + bla_{OXA-23}$	*armA*	128	32	>256	>512
AC160	$bla_{OXA-51} + bla_{OXA-23}$	*armA*	64	32	>256	>512
L25	$bla_{OXA-51} + bla_{OXA-23}$	-	32	32	0.5	>512
L26	$bla_{OXA-51} + bla_{OXA-23}$	*armA*	64	32	>256	>512
L27	$bla_{OXA-51} + bla_{OXA-23}$	*armA*	64	32	>256	>512
L28	$bla_{OXA-51} + bla_{OXA-23}$	*armA*	64	32	>256	>512
L29	$bla_{OXA-51} + bla_{OXA-23}$	*armA*	64	32	>256	>512
L12	$bla_{OXA-51} + bla_{OXA-23}$	-	32	32	16	>512
L14	$bla_{OXA-51} + bla_{OXA-23}$	-	32	32	0.5	>512
L20	$bla_{OXA-51} + bla_{OXA-23}$	*armA*	128	32	>256	>512
L21	$bla_{OXA-51} + bla_{OXA-23}$	*armA*	128	32	>256	>512
L23	$bla_{OXA-51} + bla_{OXA-23}$	*armA*	128	32	>256	>512
